# The design and growth of peanut-like CuS/BiVO_4_ composites for photoelectrochemical sensing†

**DOI:** 10.1039/d0ra01307b

**Published:** 2020-04-14

**Authors:** Yang Yang, Junting Liang, Wenwen Jin, Yingyue Li, Menghui Xuan, Shijie Wang, Xiaoqian Sun, Chuanliang Chen, Jianhua Zhang

**Affiliations:** Clinical Bioinformatics Experimental Center, Henan Provincial People's Hospital, People's Hospital of Zhengzhou University Zhengzhou Henan 450003 China henanccl@163.com; Department of Neurosurgery, Zhumadian Central Hospital Zhumadian 463000 China; Medical Engineering Technology and Data Mining Institute of Zhengzhou University Zhengzhou Henan 450000 China petermails@zzu.edu.cn

## Abstract

In this study, the CuS/BiVO_4_-*X* (where *X* represents the mass percentage of CuS associated with CuS/BiVO_4_; *X* = 2%, 5% and 7%) p–n heterostructures were fabricated using a two-step hydrothermal method. The structural and morphological features were ascertained in great detail using several physical characterization processes. According to the results of the photoelectrochemical (PEC) experimental processes, the PEC properties of CuS/BiVO_4_-5% were much more obvious as compared to those of pure BiVO_4_, CuS and CuS/BiVO_4_-*X*. Moreover, the photoluminescence (PL) and UV-vis diffuse reflection spectra (DRS) affirmed that the CuS/BiVO_4_-5% demonstrates an excellent capacity for absorbing visible light and low electron recombination rate as compared with the other composites. Accordingly, PEC sensors with CuS/BiVO_4_-5% were fabricated for the detection of dopamine (DA) and bisphenol A (BPA) with outstanding selectivity and stability. For DA, it implied a broad linear range from 0.01–10 μM and 10–120 μM, and for BPA, the broad linear range was 0.01–90 μM. Thus, the PEC sensor has significant potential application when it comes to DA and BPA detection.

## Abstract 2

In this study, the CuS/BiVO_4_-*X* (where *X* represents the mass percentage of CuS associated with CuS/BiVO_4_; *X* = 2%, 5% and 7%) p–n heterostructures were fabricated using a two-step hydrothermal method.

## Graphical abstract

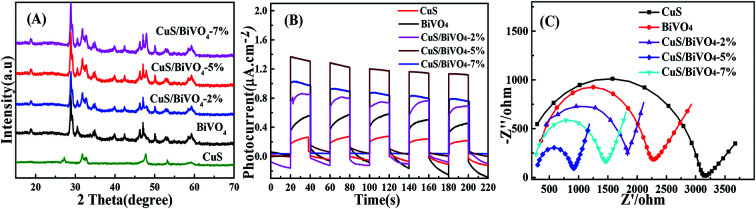

## Introduction

Bisphenol A (BPA, 2,2′-bis(4-hydroxyphenyl)propane) is a characteristic environmental incretin-disrupting element that can adversely affect multiple systems in humans and cause Parkinson's disease (PD) by inducing oxidative stress and inflammation.^1–3^ Moreover, the basic principle of treating this disease is to increase the content of dopamine (DA) in the substantia nigra.^4^ Therefore, it is important to adopt a fast and feasible method to detect the levels of DA and BPA.^5,6^

Many different approaches have been adopted to detect DA and BPA, such as fluorescence capillary electrophoresis, electrochemical and photoelectrochemical (PEC).^7–9^ To be specific, PEC analysis, with high sensitivity and ideal signal ratio, has drawn significant attention for its high cost-effectiveness, quick response as well as its excellent stability.^10–12^ The PEC method requires semiconductor materials with electrical and optical properties.^13,14^ Thus, for ultra-sensitive PEC approaches, semiconductor materials with easy fabrication and broad optical response should be developed.

To date, photocatalysts with a bismuth-system have found application in PEC study, including BiVO_4_, Bi_2_MoO_6_, Bi_2_WO_6_, BiPO_4_ and so on.^15–18^ Bismuth-system oxides, which are composed of 6s Bi orbitals and 2p oxygen orbitals, display outstanding electrical and optical characteristics.^19^ Moreover, they enjoy several popular strengths, *e.g.*, abundance, low toxicity, and low cost.^20^ Among them, bismuth vanadate (BiVO_4_), an n-type semiconductor, is regarded to be a photocatalyst that is full of potential due to its narrow band gap (2.4 eV),^21^ together with its wide use in photoelectrocatalytic water splitting,^22^ PEC sensors^23^ and PEC CO_2_ reduction.^24^ However, it is not efficient in energy conversion due to the rate of rapid charge recombination.^25^ The combination of other semiconductor elements and BiVO_4_ will help to improve the electron transfer efficiency and facilitate electron–hole pair separation, thus enhancing the energy conversion efficiency.^26^

In recent years, sulfide-based photocatalysts (MoS_2_, WS_2_, CdS *et al.*) have become the focus of most exploration as they enjoy a relatively narrow band gap as well as a greater range of light absorption.^27–29^ Copper sulfide (CuS), a promising p-type semiconductor, can absorb and utilize ultraviolet (UV) and visible light due to its small band gap (2.1 eV).^30,31^ The heterojunction formed between a p-type and an n-type semiconductor is capable of enhancing the ability to absorb visible light as well as facilitating the separation process of charge carriers.^32,33^ As such, we designed a complex of BiVO_4_ and CuS to improve the PEC performance.

In this paper, we adopted a two-step hydrothermal method and successfully synthesized CuS/BiVO_4_ heterojunction composites. The PEC activity was assessed by detecting DA and BPA under visible light irradiation. The results demonstrate that the CuS/BiVO_4_ composites developed greater PEC efficiency as compared with CuS and BiVO_4_, and showed outstanding selectivity, low detection limits and a wide linear range for detecting DA and BPA. According to the results, the sensors with CuS/BiVO_4_ have great potential for applications in the practical detection of DA and BPA.

## Experimental

### Reagents

All reagents were of analytical grade and were used without further purification. Bismuth nitrate pentahydrate (Bi(NO_3_)_3_·5H_2_O), ammonium metavanadate (NH_4_VO_3_), copper nitrate trihydrate (Cu(NO_3_)_2_·3H_2_O), sodium thiosulfate pentahydrate (Na_2_S_2_O_3_·5H_2_O) and ethylene glycol (EG) were purchased from Sinopharm Chemical Reagent Co. Ltd. (https://www.sinoreagent.com). Dopamine was purchased from Aladdin Chemical Reagent Co. Ltd. (https://www.aladdin-e.com). Human serum samples were purchased from the People's Hospital of Zhengzhou University, Clinical Bioinformatics Experimental Center.

### Preparation of the CuS/BiVO_4_ heterojunction

The CuS/BiVO_4_ heterojunction was prepared by a two-step hydrothermal method. To synthesize BiVO_4_, Bi(NO_3_)_3_·5H_2_O and NH_4_VO_3_ were dissolved in a mixture solution of EG and hot water. The solution was transferred into a 100 mL Teflon-lined stainless autoclave and kept at 120 °C for 12 h. The prepared BiVO_4_ powder was dispersed in distilled water by ultrasonication for 30 min, and appropriate amounts of Cu(NO_3_)_2_·3H_2_O and Na_2_S_2_O_3_·5H_2_O were added successively. After the addition of 10 mL of ethanol to the above solution under stirring, the mixture was transferred to a 50 mL Teflon-lined stainless autoclave and heated at 200 °C for 12 h. Finally, the product was cleaned and then dried at 60 °C. The CuS/BiVO_4_ heterojunction composed of different CuS contents was labeled as CuS/BiVO_4_-*X*, where *X* represents the mass percentage of CuS associated with the CuS/BiVO_4_ heterojunction. For comparison, different CuS/BiVO_4_ heterojunctions having different mass ratios of CuS (2 wt%, 5 wt% and 7 wt%) were also prepared.

### Electrochemical experiments

All electrochemical experimental processes were conducted using a three-electrode system (CHI 660E). Pt wire and a saturated calomel electrode (SCE) played the roles of the counter electrode and reference electrode, respectively. We performed electrochemical impedance spectroscopy (EIS) in the frequency range of 1 to 1 000 000 Hz in 0.1 M phosphate buffer (pH 7.0) produced by mixing the stock solutions of Na_2_HPO_4_ and NaH_2_PO_4_. The indium tin oxide (ITO) glass served as the working electrode, a xenon lamp (PLS-SXE 300, 100 mW^.^cm^−2^, *λ* ≥ 420 nm) was utilized as the light source. The ITO electrodes (10 × 15 mm) were cleaned separately with ethanol, acetone and water for 5 min. Next, 3 mg catalyst powders were dispersed in a chitosan and ethanol mixed solution (0.5 mL) for the formation of a homogeneous suspension. Subsequently, 20 μL suspensions were coated on ITO electrodes (0.5 cm^2^).

### Characterization

X-ray diffraction (XRD) patterns were obtained using a Bruker D8 Advance diffractometer with Cu Kα radiation. The X-ray photoelectron spectra were achieved with the help of an X-ray photoelectron spectroscope (XPS, ESCALAB 250Xi, Al Kα microfocus monochromator with variable spot size (30–400 μm and 5 μm step size)). Diffuse reflection spectra (DRS) were obtained for the materials, which were verified using a UV-vis spectrophotometer, with BaSO_4_ as the reference. The morphologies of the samples were characterized by scanning electron microscopy (SEM) and transmission electron microscopy (TEM).

## Results and discussion

### Choice of materials

**Fig. 1:**
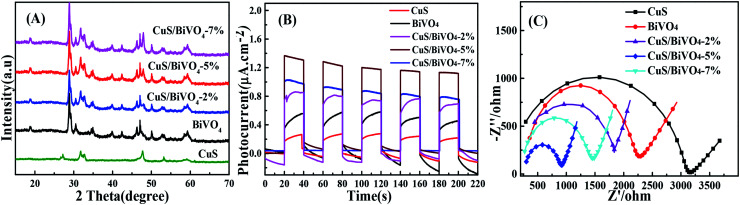
XRD spectra (A), photocurrent responses (B), and EIS (C) of the CuS, BiVO_4_ and CuS/BiVO_4_-*X* composites in 0.1 M PBS.

The phases of all synthetic materials were studied by XRD (Fig. 1A). For pure CuS, the four typical characteristic peaks around 31.78°, 32.85° and 47.94° correspond to (103), (006) and (110) of CuS (JCPDS 06-0464), while the peaks at 18.98°, 28.82°, 30.54° and 47.30° were attributed to the (110), (121), (040) and (042) lattice planes of BiVO_4_ (JCPDS No. 14-0688). For CuS/BiVO_4_, the positions of the peaks were the same even for a variety of composites, with both CuS and BiVO_4_ peaks being clearly identifiable. More importantly, with the increase in CuS, the (110) peak became more and more prominent. All results indicate that we have successfully synthesized CuS/BiVO_4_.

In order to conduct further evaluation of the PEC performances of CuS, BiVO_4_ and CuS/BiVO_4_-*X*, photocurrent densities of all materials were determined under visible light irradiation in 0.1 M PBS as shown in Fig. 1B. The photocurrent densities of all the materials were of the order CuS/BiVO_4_-5% > CuS/BiVO_4_-7% > CuS/BiVO_4_-2% > BiVO_4_ > CuS. These results show that CuS can efficiently improve the absorption of visible light and separate electron–hole pairs; excessive CuS will affect the capability of materials in absorbing visible light, thus reducing the PEC of the materials.^34^

Electrochemical impedance spectroscopy (EIS) was conducted to evaluate the electron transfer kinetics (Fig. 1C). The EIS was performed in 0.1 M PBS, which consisted of extruded semi-circular portions and linear portions. The electron transfer resistance (*R*_ct_) was quantified based on the semicircle diameter, and it was ranked as CuS > BiVO_4_ > CuS/BiVO_4_-2% > CuS/BiVO_4_-7% > CuS/BiVO_4_-5%. Double-layer capacitance (*C*_dl_), which can be obtained from cyclic voltammetry (CV), is directly related to the electrochemical active surface area (*A*) and the scanning rate *v* in a linear relationship (*C*_dl_ ∝ *v* × *A*).^35^ Therefore, the values of *C*_dl_ can be used to indicate A. As shown in Fig. S1,† the *C*_dl_ values were ranked CuS (0.29 mF cm^−2^) < BiVO_4_ (0.58 mF cm^−2^) < CuS/BiVO_4_-2% (0.72 mF cm^−2^) < CuS/BiVO_4_-7% (1.31 mF cm^−2^) < CuS/BiVO_4_-5% (1.49 mF cm^−2^). These results are consistent with EIS. All the results indicate that CuS/BiVO_4_-5% has rapid electron transfer capability, the best electrical conductivity and a large electrochemical active area, which makes it a potential active material. Based on the results shown above, CuS/BiVO_4_-5% was selected for further investigation.

### Physical characterization

**Fig. 2:**
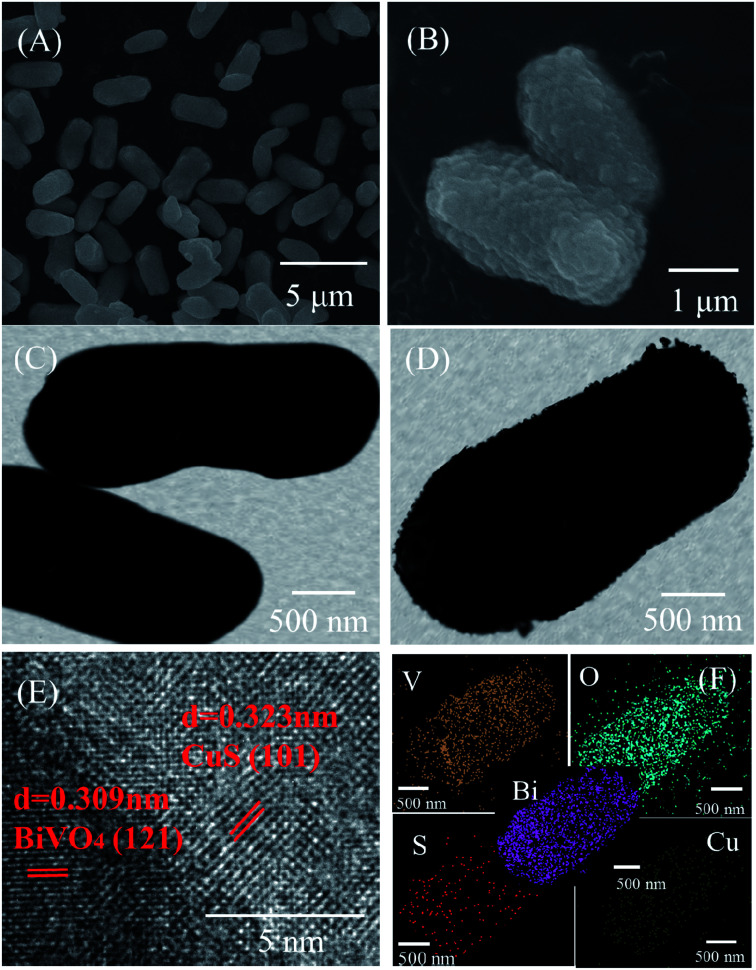
SEM images of BiVO_4_ (A) and CuS/BiVO_4_-5% (B). TEM images of BiVO_4_ (C) and CuS/BiVO_4_-5% (D). (E) HR-TEM images of CuS/BiVO_4_-5%. (F) STEM-EDX mapping images of CuS/BiVO_4_-5%.

SEM, TEM and HRTEM analysis clearly defined the morphology of the pure BiVO_4_ and CuS/BiVO_4_-5% composite. The results are shown in Fig. 2. Pure BiVO_4_ particles had the shape of a peanut with a smooth surface (Fig. 2A and C). As compared to the pure BiVO_4_, the surface of CuS/BiVO_4_-5% is rough (Fig. 2B and D). The CuS/BiVO_4_-5% composite structure was further verified by HRTEM analysis. As shown in Fig. 2E, the lattice fringes of 0.309 and 0.323 nm correspond to the (121) planes of BiVO_4_ and the (101) planes of CuS, respectively, which suggests the coexistence of BiVO_4_ and CuS in the composites. Fig. 2F shows the STEM-EDX mapping images of CuS/BiVO_4_-5%, which indicates that the Bi, V, O, Cu and S elements in the composite surface are uniformly distributed. The results further show that CuS/BiVO_4_-5% is composed of BiVO_4_ and CuS, which conforms to the XPS analysis.

**Fig. 3:**
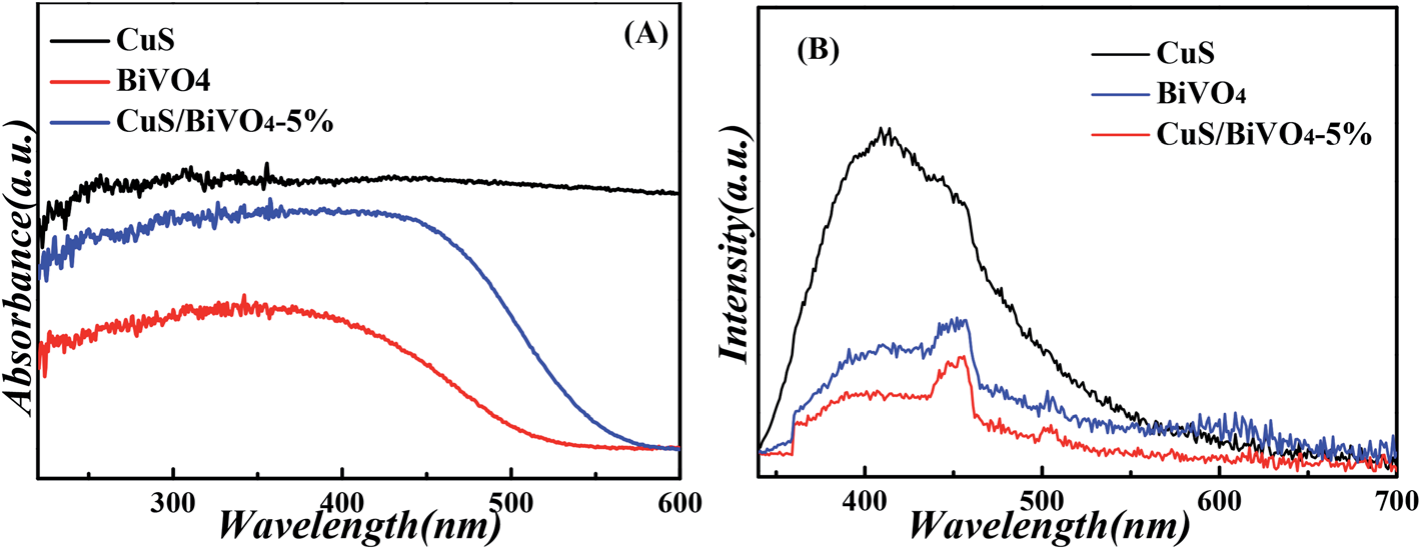
UV-vis diffuse reflectance spectra (A), and PL spectra (B) of the CuS, BiVO_4_ and CuS/BiVO_4_-5% composites.

We studied the absorption properties of BiVO_4_, CuS and CuS/BiVO_4_-5% by UV-vis DRS as shown in Fig. 3A. The pure CuS exhibited strong ultraviolet and visible absorption, while the pure BiVO_4_ showed the absorption edge at approximately 550 nm. Furthermore, the visible absorption ability of CuS/BiVO_4_ was significantly higher as compared to BiVO_4_. All these can be attributed to the inner absorption of CuS. Based on the fundamental idea of electronegativity,^36^ the band gap energy was calculated using eqn (1):

1 *αhν* = *A*(*νh* − *E*_g_)^*n*/2^

where *n* is the optical transition type, BiVO_4_ is a not direct semiconductor and the value of *n* is 4. A is a constant, *ν* represents the incident light frequency, *h* is Planck's constant, and *α* is the absorption coefficient. Fig. S2† suggests that the *E*_g_ energy gap of BiVO_4_ is 2.52 eV. CuS refers to a direct semiconductor, so *n* is 1.^32^ Accordingly, the band gap of CuS was nearly 2.26 eV. Next, the ability of synthetic materials for separating holes and electrons was studied based on PL spectra. Fig. 3B suggests that CuS/BiVO_4_-5% has the lowest intensity as compared with CuS and BiVO_4_, suggesting that CuS/BiVO_4_-5% has a low electron recombination rate.^16^ According to the discussion above, CuS/BiVO_4_-5% displayed terrific PEC performance.

### Photoelectrochemical sensor

**Fig. 4:**
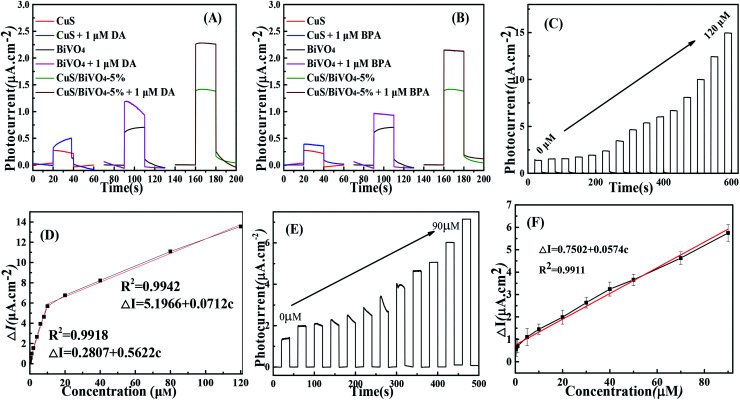
Photocurrent responses of the CuS, BiVO_4_ and CuS/BiVO_4_-5% composites with and without 1 μM DA (A) and 1 μM BPA (B). (C) Photocurrent responses of CuS/BiVO_4_-5% toward DA with increasing concentrations of DA. (D) The relevant calibration plots of the DA concentration. (E) Photocurrent responses of CuS/BiVO_4_-5% toward BPA with increasing concentrations of BPA. (F) The relevant calibration plots of the BPA concentration in 0.1 M PBS at 0 V *vs.* SCE as excited by visible light.

Fig. 4A and B suggests the photocurrent performance of CuS, BiVO_4_ and CuS/BiVO_4_-5% at 0 V *vs.* SCE with the addition of 1 μM DA and BPA under visible light excitation in 0.1 M PBS. The photocurrent of all synthetic materials increased with the addition of DA and BPA. Meanwhile, the photocurrent of CuS/BiVO_4_-5% was larger than that of BiVO_4_, since the p–n heterostructure improved the PEC efficiency for DA and BPA to a large extent.

To further evaluate the PEC performance of CuS/BiVO_4_-5% for DA and BPA, the photocurrent change was ascertained at many different concentrations of DA and BPA. By the respective addition of DA, the photocurrent response increased significantly as shown in Fig. 4C. A linear relationship was found between the concentration of DA and photocurrent (Fig. 4D), and it displays two linear associations based on the sensor of CuS/BiVO_4_-5% for DA. From 0.01–10 μM, the linear regressing equation is Δ*I* = 0.2807 + 0.5622*c* (*R*^2^ = 0.9918), ranging from 10 to 120 μM; the linear association is Δ*I* = 5.1966 + 0.0712*c* (*R*^2^ = 0.9942). The detection limit of the sensor is 3.4 nM (S/N = 3).

Experiments for BPA detection were performed in PBS (Fig. 4E and F). On increasing the BPA concentration in PBS, the photocurrent increased and the as-prepared CuS/BiVO_4_-5% photoelectrode detected BPA with a linear range from 0.01 μM to 90 μM. The linear regressing equation is Δ*I* = 0.7052 + 0.0574*c* (*R*^2^ = 0.9911) with a detection limit (S/N = 3) of 7 nM. In Table 1 a comparison is made of CuS/BiVO_4_-5% with other DA and BPA sensors in the literature. It is undeniable that the CuS/BiVO_4_-5% sensor outperforms the other DA and BPA sensors in several fields.

**Table 1:** Comparison of different methods for detecting DA and BPA

Method	Materials	Analyst	Linear range (μM)	LOD	Ref.
Fluorescent	MoS_2_	DA	0.1–100	10 nM	42
Electrochemical	CuO/CN	DA	0.2–78.7	0.06 μM	4
Electrochemical	RGO-ZnO	DA	1–70	0.33 μM	43
PEC	SnSe NSs	DA	0.01–10	3 nM	7
PEC	WO_3_	DA	53–80, 85–155	0.3 μM	44
PEC	CuS/BiVO_4_	DA	0.01–10	3.4 nM	This work
			10–120		
Fluorescence polarization immunoassay	4,4-Bis(4-hydroxyphenyl)valeric acid	BPA	0.087–3.5	8.7 nM	45
PEC	TiO_2_/Au NTAs	BPA	0.1–28.9	0.047 μM	9
PEC	ZnPc/TiO_2_NRs	BPA	0.047–52.1	8.6 nM	46
PEC	CuS/BiVO_4_	BPA	0.01–90	7 nM	This work

**Fig. 5:**
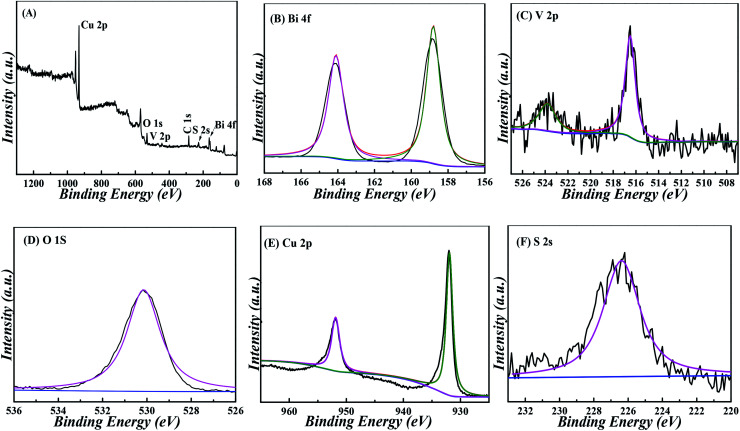
XPS spectra of CuS/BiVO_4_-5% composites: (A) survey, (B) Bi 4f, (C) V 2p, (D) O 1s, (E) Cu 2p and (F) S 2s.

**Fig. 6:**
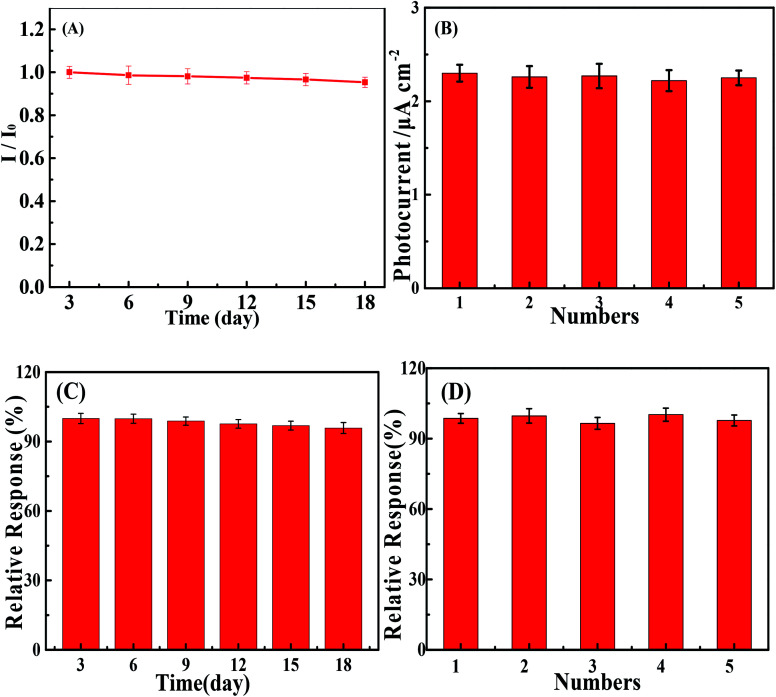
Stability and reproducibility tests of the PEC sensor based on CuS/BiVO_4_-5% towards DA (A and B) and BPA (C and D).

By monitoring the photocurrent of the repeated photoexcitation over 600 s, the CuS/BiVO_4_-5% stability was also checked (Fig. S3†). The response photocurrent of CuS/BiVO_4_-5% remained at 95.3% and 95.8% of its initial value towards 1 μM DA and 1 μM BPA within 18 days (Fig. 6A and C). XPS of CuS/BiVO_4_-5% before the stability test was also evaluated, as shown in Fig. 5A. For Bi 4f, O 1s, V 2p, Cu 2p and S 2s, the high resolution is shown in Fig. 5B–F. Fig. 5B shows two core peaks of the Bi 4f XPS spectra situated at 158.8 eV and 164.2 eV. The peaks at 164.2 eV and 158.8 eV are attributed to Bi 4f_5/2_ and Bi 4f_7/2_ in normal Bi^3+^.^37^ The two 2p_3/2_ (516.5 eV) and 2p_1/2_ (523.9 eV) peaks of biological iodine correspond to V^5+^ (Fig. 5C).^38^ The O 1s peak is shown in Fig. 5D, and the O 1s peak at 530.46 eV is due to the lattice oxygen.^39^ Cu 3p_1/2_ at 951.8 eV and Mo 3p_3/2_ at 931.9 eV are matched to Cu^2+^ in Fig. 5E.^40^ In Fig. 2F, the 2s peak at around 226.1 eV was attributed to S 2s, which strongly proved how CuS^41^ was formed. Fig. S4† shows that the XRD and XPS of CuS/BiVO_4_-5% after the stability test. All the results show that CuS/BiVO_4_-5% has the best stability. The reproducibility of CuS/BiVO_4_-5% was tested by detecting 1 μM DA and BPA using 5 parallel electrodes (Fig. 6B and D), and the photocurrent did not exhibit any significant variation, indicating its good reproducibility.

The selectivity of the PEC sensor based on CuS/BiVO_4_-5% towards DA and BPA is shown in Fig. S5;† the photocurrent responses of interferences can be ignored, implying that CuS/BiVO_4_-5% can selectively and effectively detect DA and BPA. To verify the practical reliability of the DA and BPA sensor that has been prepared, we used lake water containing BPA, and human blood serum containing DA as convincing samples. As shown in Table 2, for DA, the RSD values are less than 4.21%, and the recoveries are 99.80–101.0%. For BPA, the RSD values are less than 3.19%, and the recoveries are 98.00–100.12%. According to the mentioned results, the sensor based on CuS/BiVO_4_-5% is reliable for the detection of DA and BPA in actual samples.

**Table 2:** PEC detection of DA and BPA in real samples

Real samples	Added (μM)	Found (μM)	Recovery (%)	RSD (%)
Human blood serum (DA)	1.00	1.01	101.00	1.31
	5.00	4.95	99.90	2.24
	20.00	19.96	99.80	3.11
	50.00	49.92	99.84	3.52
	100.00	99.96	99.96	4.21
Lake water (BPA)	1.00	0.98	98.00	1.01
	5.00	4.98	99.60	1.95
	20.00	20.02	100.10	2.33
	40.00	40.05	100.12	2.71
	80.00	80.09	100.11	3.19

### Proposed mechanism of PEC

To explore the mechanism of PEC sensors based on CuS/BiVO_4_, the valence band energy (*E*_VB_) and the conduction band energy (*E*_CB_) of BiVO_4_ and CuS were investigated with the aid of the following equation:^47^

2 *E*_VB_ = *X* − *E*_e_ + 0.5*E*_g_

3 *E*_CB_ = *E*_VB_ − *E*_g_

**Fig. 7:**
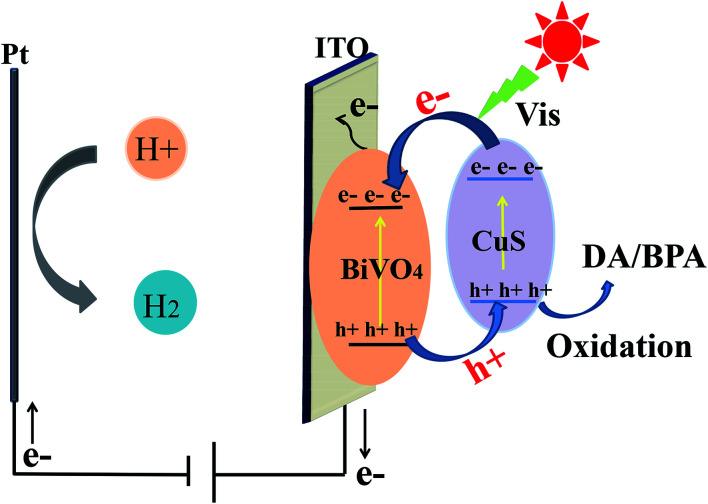
PEC mechanism of CuS/BiVO_4_ for the detection of DA and BPA.

The value of *E*_e_ was almost 4.5 eV, indicating the energy of free the electrons at the scale of hydrogen. *X* represents the semiconductor electronegativity, and *X* was 6.04 eV and 5.29 eV for BiVO_4_ and CuS, respectively. The VB edges of BiVO_4_ and CuS were +2.8 eV and +1.92 eV, respectively. Besides, we determined the CB edge potentials of BiVO_4_ and CuS as +0.28 eV and −0.34 eV, respectively. As shown in Fig. 7, the migrating process of photogenerated electrons occurs from the CB of CuS to the CB of BiVO_4_ and the holes are transferred from the VB of BiVO_4_ to the VB of CuS due to the dynamic principle, which could help divide the photogenerated electron–hole pairs to effectively utilize the oxidization of holes. By the same token, the holes will be utilized to oxidize DA and BPA.

## Conclusion

In summary, we have designed a CuS/BiVO_4_ composite *via* a two-step hydrothermal method. CuS/BiVO_4_-5% exhibited superior PEC properties for DA and BPA as compared with pure BiVO_4_, CuS and CuS/BiVO_4_-*X*. The CuS content had an impact on the PEC efficiency of CuS/BiVO_4_. The PEC efficiency and stability of CuS/BiVO_4_ can be attributed to the appropriate CuS content as well as the influential electron–hole pair separation. The study indicates that the sensor based on CuS/BiVO_4_-5% is sure to possess great application prospects for DA and BPA PEC detection.

## Conflicts of interest

There are no conflicts to declare.

## Supplementary Material

RA-010-D0RA01307B-s001
