# Supplementary material for: The design and growth of peanut-like CuS/BiVO4 composites for photoelectrochemical sensing
Source: RSC Adv. 2020 Apr 14;10(25):14670–8. doi: 10.1039/d0ra01307b (PMC9051948; doi:10.1039/d0ra01307b)
Supplement: RA-010-D0RA01307B-s001 [file RA-010-D0RA01307B-s001.pdf]

Electronic Supplementary Material (ESI) for RSC Advances.
This journal is © The Royal Society of Chemistry 2020

The design and growth of peanut-like CuS/BiVO$_4$ composite for photoelectrochemical sensing

Yang Yang[1, 2#], Junting Liang[1#], Wenwen Jin[3], Yingyue Li[3], Menghui Xuan[3], Shijie Wang[3], Xiaoqian Sun[3], Chuanliang Chen[1*], Jianhua Zhang[3*]

[1] Clinical Bioinformatics Experimental Center, Henan Provincial People's Hospital, People's Hospital of Zhengzhou University, Zhengzhou, Henan, 450003, China;

[2] Department of Neurosurgery, Zhumadian Central Hospital, Zhumadian 463000, China;

[3] Medical Engineering Technology and Data Mining Institute of Zhengzhou University, Zhengzhou, Henan, 450000, China.

[*] Email of Corresponding Author: petermails@zzu.edu.cn (Jianhua Zhang); henanccl@163.com (Chuanliang Chen).

[#] Co first author.

Table of contents

1. CV curves of the as-prepared catalysts

2. The Eg gap of $BiVO_4$ and CuS

3. Stability tests of PEC sensor based on CuS/$BiVO_4$-5% towards DA and BPA

4. XRD pattern and XPS spectra of CuS/$BiVO_4$-5% after stability test

5. The selectivity of PEC sensor based on CuS/$BiVO_4$-5% towards DA and BPA

6. Contents of different elements analyzed by XPS

1. CV curves of the as-prepared catalysts

Fig. S1 shows the CV curves of CuS/$BiVO_4$-5%, respectively, at different scan rates (from 10 to 100 mV·s-1). Figure S1(B) indicate the Cdl values of all materials, and it will be ranked as: CuS (0.30 mF·cm^{-2}) < $BiVO_4$ (0.59 mF·cm^{-2}) < CuS/$BiVO_4$-2% (0.73 mF·cm^{-2}) < CuS/$BiVO_4$-7% (1.32 mF·cm^{-2}) < CuS/$BiVO_4$-5% (1.50 mF·cm^{-2}). The results show that the surface roughness of CuS/$BiVO_4$-5% is higher than others.

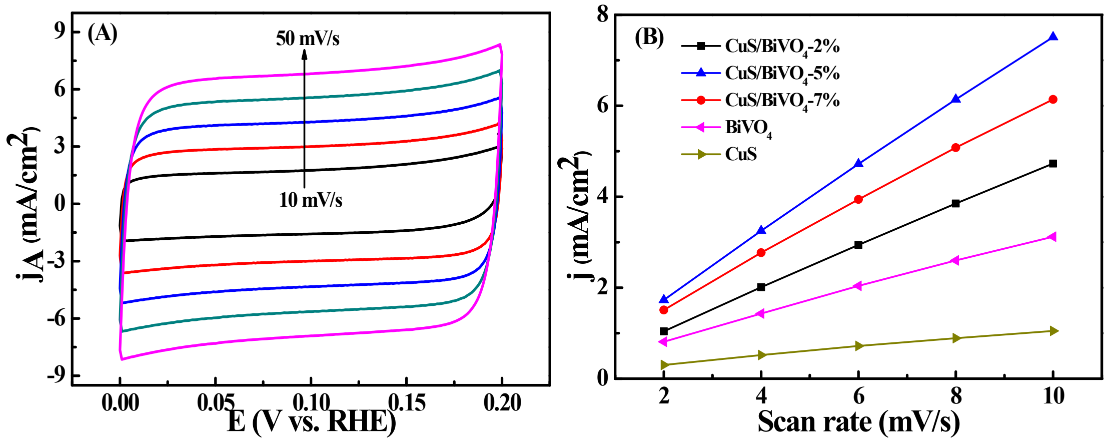

Fig. S1. (A) CV curves of CuS/$BiVO_4$-5%, respectively, at different scan rates. (B) Cdl for CuS, $BiVO_4$ and CuS/$BiVO_4$-X, respectively.

2. The Eg gap of $BiVO_4$ and CuS

On the basis of the basic electronegativity concept, the Eg gap energy of $BiVO_4$ is 2.52 eV and the corresponding band gap of CuS was about 2.26 eV (Fig. S2).

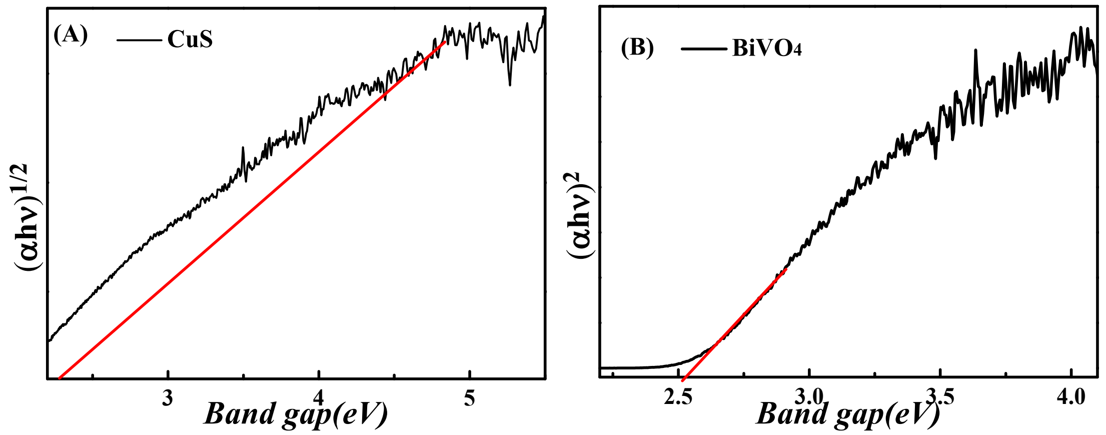

Fig. S2 Plots of $(Ahv)^{1/n}$ vs. photon energy (hv) for CuS (A) and $BiVO_4$ (B).

3. Stability tests of PEC sensor based on $CuS/BiVO_4$-5% towards DA and BPA

The stability of CuS/BiVO4-5% was also checked by monitoring the photocurrent of repeated photoexcitation over 600 s. And the photocurrent did not show any obvious change, indicating its excellent stability.

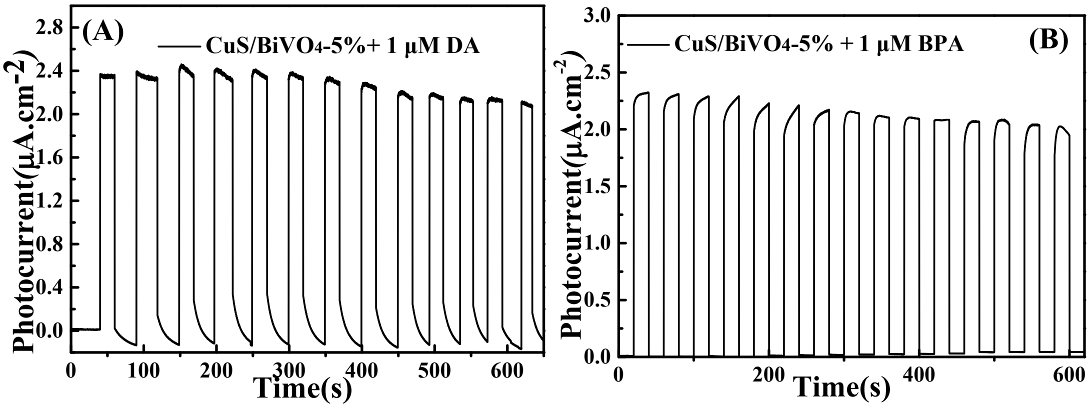

Fig. S3 Stable photocurrent response curve of the $CuS/BiVO_4$-5% in the presence of 1 μM DA (A) and 1 μM BPA (B) in 0.1 M PBS at 0 V vs SCE with visible light excitation.

4. XRD pattern and XPS spectra of CuS/$BiVO_4$-5% after stability test

Fig. S4(A) shows XRD pattern of CuS/$BiVO_4$-5% after stability test. Obviously, the peaks are almost the same as those before stability test, matches well with the standard cards. Meanwhile, XPS spectras of Bi 4f, V 2p, O 1s, Cu 2p and S 2s for CuS/$BiVO_4$-5% after stability test are almost the same as those before stability test (Fig. S4(B-F)). These results indicate CuS/$BiVO_4$-5% has an excellent stability.

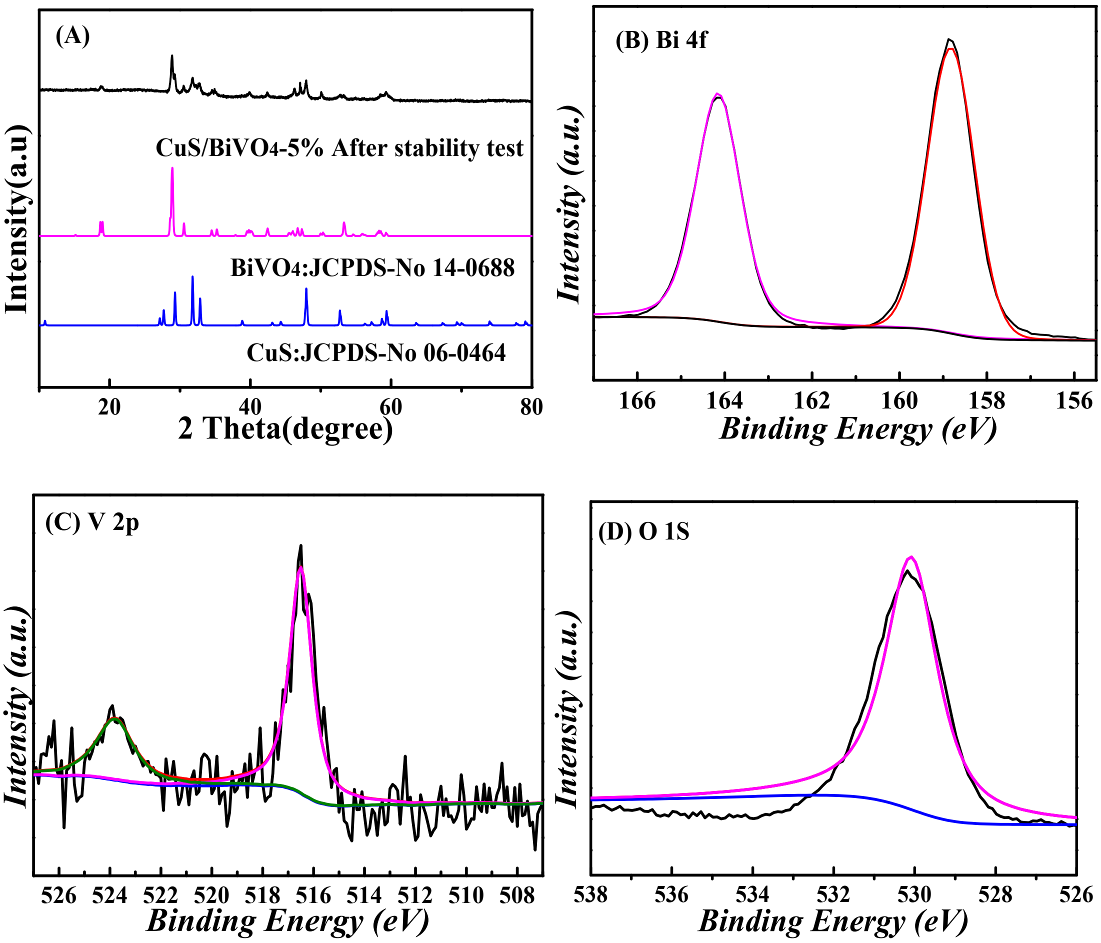

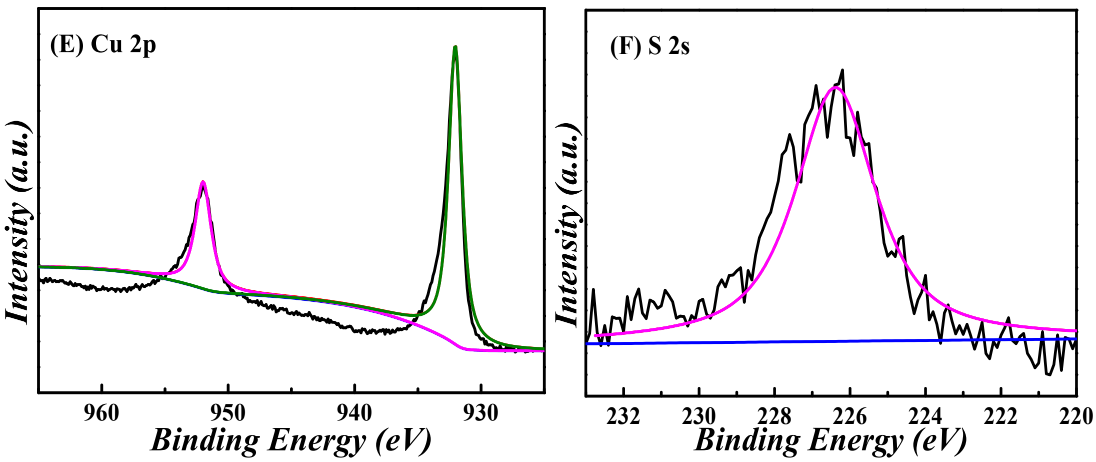

Fig. S4. XRD (A) and XPS (B-F) of $CuS/BiVO_4$-5% after stability test.

5. The selectivity of PEC sensor based on CuS/BiVO$_4$-5% towards DA and BPA

In order to investigate the selectivity of sensor, we inject a wide range of co-existing ions. Fig. S5 suggests that the CuS/BiVO$_4$-5% exhibits a good selectivity.

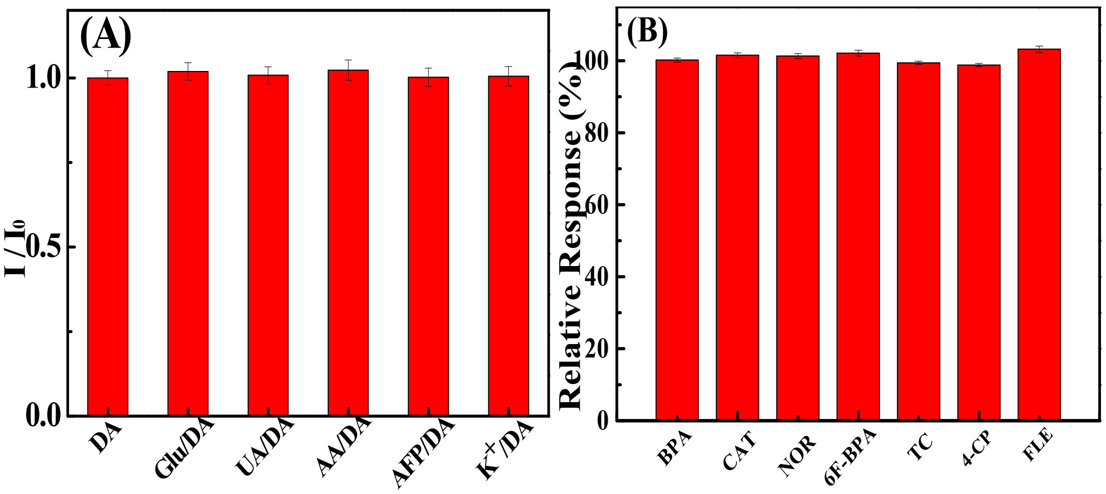

Fig. S5 The selectivity of PEC sensor based on CuS/BiVO$_4$-5% towards DA (A) and BPA (B) in 0.1 M PBS at 0 V vs SCE with visible light excitation.

6 Contents of different elements analyzed by XPS

The contents of different elements were analysized by XPS. The results are listed in Table S1. As we can see, the results analyzed by XPS agree well with with the original feed ratios.

Table S1 Contents of different elements analyzed by XPS.

Sample	C atom %	Cu atom %	S atom %	Bi atom %	V atom %	O atom %
$CuS/BiVO_4$-5%	29.77	3.02	1.54	38.06	11.25	16.36

f
